# Reducing attrition within clinical trials: The communication of retention and withdrawal within patient information leaflets

**DOI:** 10.1371/journal.pone.0204886

**Published:** 2018-10-31

**Authors:** Anna Kearney, Anna Rosala- Hallas, Naomi Bacon, Anne Daykin, Alison R. G. Shaw, Athene J. Lane, Jane M. Blazeby, Mike Clarke, Paula R. Williamson, Carrol Gamble

**Affiliations:** 1 North West Hub for Trials Methodology Research/ Clinical Trial Research Centre, Biostatistics, University of Liverpool, Liverpool, United Kingdom; 2 Clinical Trial Research Centre, Biostatistics, University of Liverpool, Liverpool, United Kingdom; 3 ConDuCT-II Hub for Trials Methodology Research, University of Bristol, Bristol, United Kingdom; 4 Centre for Public Health, Queen’s University of Belfast, Belfast, United Kingdom; Iranian Institute for Health Sciences Research, ISLAMIC REPUBLIC OF IRAN

## Abstract

**Background:**

The recruitment and retention of patients are significant methodological challenges for trials. Whilst research has focussed on recruitment, the failure to retain recruited patients and collect outcome data can lead to additional problems and potentially biased results. Research to identify effective retention strategies has focussed on influencing patient behaviour through incentives, reminders and alleviating patient burden, but has not sought to improve patient understanding of the importance of retention. Our aim is to assess how withdrawal, retention and the value of outcome data collection is described within the Patient Information Leaflets (PIL) used during consent.

**Methods:**

Fifty adult or parent PIL from a cohort of trials starting between 2009–2012 and funded by the NIHR Health Technology Assessment programme were obtained from protocols, websites or by contacting trialists. A checklist of PIL content based on Health Research Authority (HRA) and ICH GCP Guidelines was supplemented with retention specific questions. Corresponding protocols were also evaluated to cross reference trial specific procedures with information communicated to patients.

**Results:**

PIL frequently reiterated the patient’s right to withdraw at any time (n = 49, 98%), without having to give a reason and without penalty (n = 45, 90%). However, few informed patients they may be asked to give a withdrawal reason where willing (n = 6, 12%). Statements about the value of retention were infrequent (n = 8, 16%). Consent documents failed to include key content that might mitigate withdrawals, such as the need for treatment equipoise (n = 3, 6%). Nearly half the trials in the cohort (n = 23, 46%) wanted to continue to collect outcome data if patients withdraw. However, in 70% of PIL using prospective consent, withdrawal was described in generic terms leaving patients unaware of the difference between stopping treatment and all trial involvement. Nineteen (38%) trials offered withdrawing patients the option to delete existing data.

**Conclusions:**

Withdrawal and retention is poorly described within PIL and addressing this might positively impact levels of patient attrition, reducing missing data. Consent information is unbalanced, focussing on patient’s rights to withdraw without accompanying information that promotes robust consent and sustained participation. With many citing altruistic reasons for participation it is essential that PIL include more information on retention and clarify withdrawal terminology so patients are aware of how they can make a valuable contribution to clinical studies. There is a need to determine how retention can be described to patients to avoid concerns of coercion. Future research is needed to explore whether the absence of information about retention at the time of consent is impacting attrition.

## Introduction

The recruitment and retention of patients are acknowledged as major challenges in the delivery of clinical trials and represent key methodological research priorities [1, 2].

Research has focussed on assessing the reasons and barriers to participation and identifying effective strategies to recruit participants [3]. However, these efforts are misplaced if recruited patients are not retained or do not contribute outcome data.

Both inadequate recruitment and poor retention can lead to underpowered studies. Low levels of patient retention and the resulting missing data can also lead to a biased interpretation of treatment effect depending on the level of missing outcome data, the reasons for it and the distribution across trial arms [4].

Poor reporting makes it difficult to assess the extent of attrition within clinical trials [5–7]. Analysis of cohorts show median missing outcome data ranging between 6 and 11% of randomised participants [4, 6, 8], although individual trials have reported up to 70% losses [8]. Longitudinal studies [9] mental health studies [10, 11], and trials within minority populations are acknowledged to have particularly difficult retention issues [12, 13].

Retention issues are usually addressed within statistical analysis either by excluding missing data from the analysis population (complete case) or using imputation methods to estimate the outcome [8]. Prevention is better than cure and the FDA and EMA recommend that mitigating patient withdrawals and missing data through improving trial design and conduct should be the primary approach [9, 14].

There is currently little evidence to inform the use of effective strategies to address retention problems within trial design and conduct. Research to date has sought to influence patient behaviour through the use of incentives, reminders and minimising patient burden [15]. An alternative not explored is how to improve patient understanding of the impact of adhering to follow up schedules and data collection.

There is an ethical requirement to ensure potential participants are adequately informed about trial participation. Given the impact of attrition and the altruistic ambitions of participants, retention should be raised at consent to avoid wasting participant’s time if they can’t adhere to the trial schedule or will only continue if assigned a specific treatment. However, communicating this is challenging given concerns about coercion.

The aim of this paper is to assess how withdrawal, retention and the value of outcome data collection are described in Patient Information Leaflets (PIL) to see whether patients are adequately informed about the importance of trial retention during consent.

## Methods

### Cohort selection

The National Institute for Health and Research’s Health and Technology Assessment (NIHR HTA) programme is the largest public funder of pragmatic randomised control trials in the UK, providing a representative and easily accessible cohort with which to assess retention information. We searched the NIHR HTA online portfolio [16] to identify randomised controlled trials (RCTs) with start dates between 2009 and 2012 [16]. The start dates for the cohort ensured recruitment had started following any pilot work or delays obtaining governance approvals. The portfolio search engine was restricted to primary research with the search conducted on the 23^rd^ September 2014. This cohort was identified as part of a wider project on retention.

Online summary records were screened for eligibility, before conducting a full text review of the project protocol available on the HTA website. We included all parallel two-arm RCTs and applied the following exclusion criteria: Factorial trials; Cluster randomised trials; Crossover trials; Feasibility or pilot trials; Sub studies within a trial; Cohort (non-randomised); Papers reporting longer term follow up of trial patients; Phase 1 or 2 trials (trials described as Phase 2/ 3 have been included).

### Accessing patient information leaflets (PIL)

Adult patient information Leaflets (PIL), or parent information leaflets for paediatric trials, were obtained from protocols, trial specific websites or Clinical Trial Unit (CTU) websites. When patient information leaflets were not publically available Chief Investigators and Trial Managers were contacted to request a copy. If multiple copies were obtained for the same trial the most recent version was used.

Where consent forms were contained within the same document as the information leaflet they were not included in the content analysis. Our deliberate decision not to analyse them was based on the understanding that consent forms should not present new material, but rather act as a written record of the decision to participate based on the information received.

### Data extraction and analysis

Informed consent criteria described in ICH GCP section 4.8 [17] and Health Research Authority (HRA) guidance [18] for PIL content were used to develop a checklist of essential content. This was supplemented with additional retention questions developed by authors AK and CG. (Table 1)

**Table 1:** Checklist of retention content for PIL.

**HRA Guidelines on key PIL information:**
• Title of the study
• An invitation to participate
• A summary of involvement
• The purpose of the research
• What taking part involves
• Benefits and risks of participation
• What happens if something goes wrong
• Confidentiality of information
• The results of the study
• Who is organising and funding the study
• How patients and public have been involved in the study
• Who has reviewed the study
• Further Information and contact details
• Details of the consent process
**ICH GCP and HRA Guidelines on withdrawal content**
• Communicate that participants are free to withdraw at any time without it affecting the care they receive
• PIL should clearly define what patients are to expect when withdrawing e.g. what are they withdrawing from?
• Outline processes (where possible) for deleting data should a patient wish
**Additional retention questions developed by authors**
• Does the PIL explain that patients might be asked for a withdrawal reason?
• Does the PIL explain that patients must be willing to accept all possible trial treatments?
• Does the PIL explain the importance of retention?
• Does the PIL explain the impact of missing or deleted trial data on the ability to answer the clinical question?

Corresponding protocols were downloaded from the HTA portfolio website in order to cross reference trial specific processes with the information communicated to patients within the PIL. Researchers (AK, NB and ARH) dual reviewed protocol content and extracted any text associated with participants withdrawing from the trial, including both participant and investigator initiated decisions.

Within clinical trials the term ‘withdrawal’ can be used to describe different scenarios: patients who stop all trial involvement and patients who stop their assigned treatment but are willing to allow further data collection or follow up. We reviewed PIL and protocols for descriptions of processes for withdrawal as well as stopping treatment to ascertain whether they distinguished between these scenarios.

We use the phrase ‘premature discontinuation of treatment’ where follow up is still ongoing and ‘study withdrawal’ where patients withdraw consent for all study involvement including further data collection.

Data corresponding to the retention questions used to assess PIL content were extracted from the protocol text by AK and CG.

## Results

**Fig 1:**
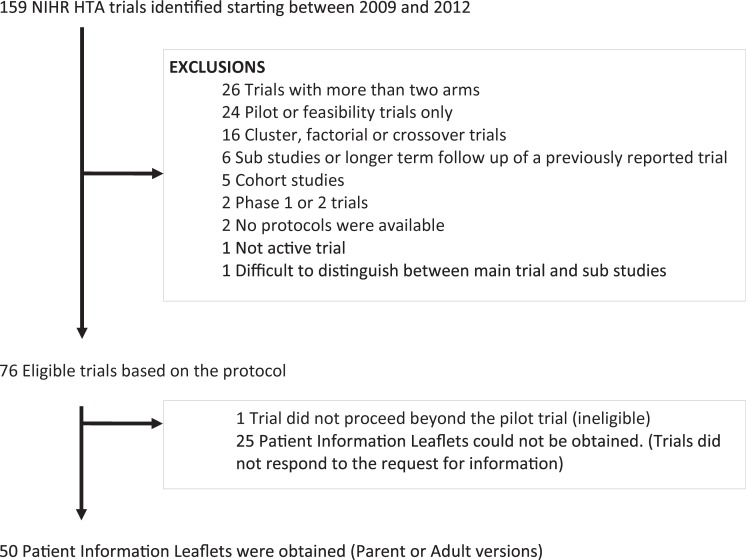
Identification of a cohort of trials.

One hundred and fifty-nine HTA funded studies were identified through their website, of which 75 were eligible for inclusion. Fifty (66.7%) PIL and their corresponding protocols were obtained for analysis (Fig 1).

The cohort covered a range of health research categories, with the greatest number of trials in mental health (n = 7, 14%) and oral gastrointestinal (n = 7, 14%). The most frequent interventions were drug (n = 13, 26%), device (n = 11, 22%) and surgical (n = 10, 20%). Forty seven of the PIL evaluated used prospective consent, with three trials using deferred consent (Table 2).

**Table 2:** Characteristics of included trials.

	No. of trials (%) n = 50
**Health Category (HRCS)**	
Mental Health	7 (14%)
Oral and gastrointestinal	7 (14%)
Injuries and accidents	5 (10%)
Reproductive Health	5 (10%)
Stroke	4 (8%)
Neurological	3 (6%)
Renal and Urogenital	3 (6%)
Respiratory	2 (4%)
Skin	2 (4%)
Blood	1 (2%)
Cancer	1 (2%)
Cardiovascular	1 (2%)
Congenital Disorders	1 (2%)
Ear	1 (2%)
Eye	1 (2%)
Generic	1 (2%)
Infection	1 (2%)
Inflammatory	1 (2%)
Musculoskeletal	1 (2%)
Metabolic	1 (2%)
Other	1 (2%)
**Intervention:**	
Drug	13 (26%)
Device	11 (22%)
Surgical	10 (20%)
Behavioural	6 (12%)
Physical	5 (10%)
Other	5 (10%)
**Other:**	
One off intervention	15 (30%)
Deferred consent only	3 (6%)

**Fig 2:**
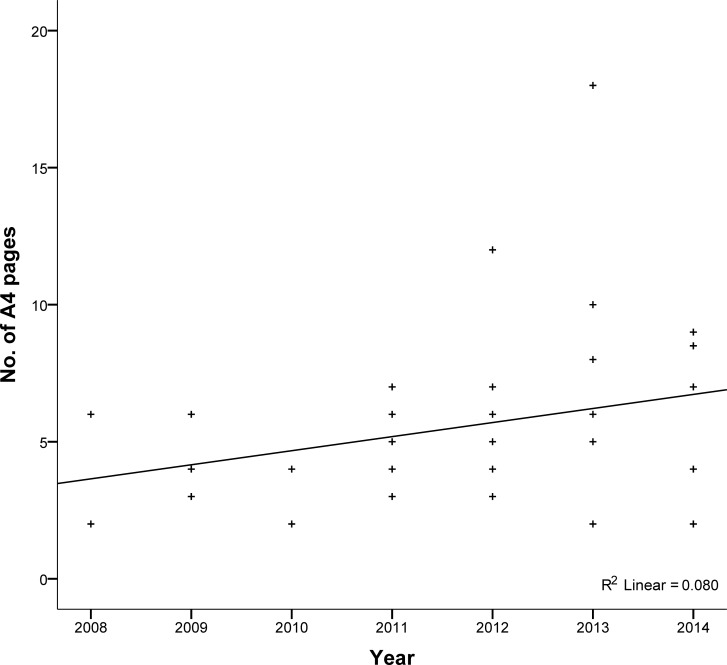
The length of patient information leaflets (n = 46^a^). ^**a**^ The date on four patient information leaflets could not be identified.

Patient Information Leaflets were written between 2008 and 2014 and protocols between 2010 and 2014. There was marked variation in the length of PIL from two to eighteen A4 pages (median 5, IQR 2). There was a tendency for the length of PIL to increase over the years (Fig 2). Protocols ranged from 14 to 196 A4 pages including appendices (median 35, IQR 33).

All PIL contained key content mandated by the HRA and ICH GCP, explaining trial treatments and procedures (Table 3). Communication of the trials duration (n = 48, 96%), and the associated risks (n = 47, 94%) and benefits (n = 46, 92%) were frequently covered within the PIL cohort. Only 70% (n = 35) explained the probability of receiving each treatment. Few explicitly stated that patients or doctors could not choose the treatment (n = 13, 26%), although this was often implied (n = 45, 90%)

**Table 3:** Frequency of trial information communicated within PIL and protocols.

	No. (%) of trials n = 50
**Patients (PIL analysis)**	
The trial treatment(s)	50 (100%)
The probabilities of getting each treatment	35 (70%)
Does the PIL communicate (either explicitly or implicitly) that you or your doctor will not determining treatment?	45 (90%)
Does the PIL explicitly describe that the treatment will not be determined by you or your doctor	13 (26%)
The trial procedures to be followed, including all invasive procedures	50 (100%)
The subject’s responsibilities/ What taking part involves	50 (100%)
The expected duration of the subject’s participation in the trial	48 (96%)
The reasonably foreseeable risks or inconveniences to the subject and, when applicable, to an embryo, foetus, or nursing infant.	47 (94%)
The reasonably expected benefits. When there is no intended clinical benefit to the subject, the subject should be made aware of this	46 (92%)
**Patients (Retention content in PIL)**	
PIL state that patients must be willing to have any treatment to enter the trial ^a^	3 (6%)
Indirect or direct comment about the importance of retention	8 (16%)
The PIL describes the importance of follow up whilst receiving treatment	2 (4%)
The PIL describes the importance of follow up whilst if the allocated treatment is not received	2 (4%)
The PIL describe the importance of follow up whilst if the allocated treatment is stopped	4 (8%)
The PIL makes a distinction between withdrawal from treatment and withdrawal from follow up ^a^	14 (30%)
**Clinicians/ Research sites (Protocol analysis)**	
Protocol contains information on withdrawal	43 (86%)
Protocol makes a distinction between withdrawal from treatment and withdrawal from follow up ^a^	28 (60%)
States that the trial wants to collect follow up data if a patient withdraws	23 (46%)

^a^n = 47.

Three trials with deferred consent excluded (two trials recruiting adults and one with no age limits).

*“Neither you nor your doctor will be able to decide which method of feeding you receive”*. *(PIL 5, explicit)*

*“The allocation process will be done by a computer and will be done purely by chance” (PIL 4*, *Implicit)*

Only three trials, all of which had surgical interventions, explained patients should be willing to accept either treatment if they wished to take part in the trial.

“To take part you must be prepared to have either operation” (PIL 36)

“To take part in this study you must be happy to be in either of the treatment groups” (PIL 2)

“As the treatment is always chosen at random this also means that if you have a preference for the treatment you’d like to receive then you won’t be able to take part in the trial” (PIL 45)

### Statements about patient withdrawal

Forty three (86%) trial protocols contained information on patient withdrawal with content ranging from only confirming patient’s right to withdraw from a trial to describing trial specific withdrawal processes (Table 3).

**Fig 3:**
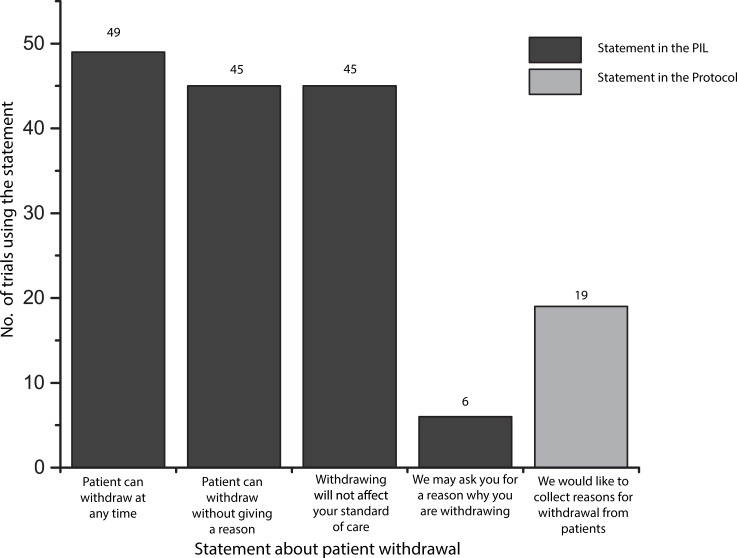
Frequency of communication about patient withdrawal within PIL and protocols (n = 50).

All PIL made reference to the patients right to ‘withdraw’ from the trial after initial consent, frequently reiterating their ‘right to withdraw from the trial at any time’ (n = 49, 98%), ‘without penalty or it affecting their standard care’ (n = 45, 90%) and ‘without the need to give a reason’ (n = 45, 90%) (Fig 3). Trials were poor at communicating that patients may be asked for a reason why they are withdrawing. Nineteen trial protocols stated that they would like to collect a reason for withdrawal where patients were willing to provide one. However, only six trials (12%) communicated this within the Patient Information Leaflets. Where this was communicated to patients in the PIL, this was often described as a means of giving feedback to help improve the trial:

“You do not have to give a reason why you want to stop, but this information can be helpful because it can help us identify problems with the study and areas for improvement” (PIL 9)

### Definitions of patient withdrawal across PIL and protocols

Of the 47 trials using prospective consent, 28 (60%) trial protocols and only 14 (30%) PIL sought to differentiate between the different types of ‘withdrawal’ and address the ambiguous terminology. Only 10 trials distinguished between premature discontinuation of treatment and study withdrawal in both their PIL and Protocol (Table 4). Twenty three protocols (46%) explicitly stated that they wanted to continue to collect follow up data if a patient stopped receiving their allocated treatment. However, only two PIL (4%) explained the importance of follow up if the assigned treatment is not received and only four trials (8%) described the importance of follow up if patients stop treatment.

**Table 4:** Consistency of retention communication between trial documents.

Trial Processes/ Trial Approach	Documents communicating trial processes No of trials (%), n = 50			
	Inconsistent		Consistent	
	PIL Only	Protocol only	Both	Neither
Make a distinction between withdrawal from treatment and all trial processes including follow up ^a^	4 (9%)	18 (38%)	10 (21%)	15 (32%)
Describe processes for handling data collected before withdrawal	9 (18%)	7 (14%)	15 (30%)	19 (38%)
Offer patients the option of deleting data collected before withdrawal	9 (18%)	5 (10%)	5 (10%)	31 (62%)
Describe options around stopping some aspects of follow up	5 (10%)	9 (18%)	3 (6%)	33 (66%)
a) Describe options for continuing with routine data collection only	3 (6%)	7 (14%)	2 (4%)	38 (76%)
b)Describe options for collecting data from national records	3 (6%)	3 (6%)	0 (0%)	44 (88%)
c)Describe options around trial specific data collection e.g. questionnaires	2 (4%)	4 (8%)	0 (0%)	44 (88%)

^a^n = 47.

Three trials with deferred consent excluded.

Some PIL clearly described the option of stopping study treatments whilst continuing within the trial:

“If you decide to stop taking part in the study treatments, it would be helpful if you would keep in contact with us to complete the follow-up questionnaires and let us know your progress. You don’t have to do this and can choose to stop taking part in the whole study if you wish” (PIL 9)

“If you do withdraw from study treatment, we would like to continue to follow up and collect data as part of your normal clinic visit. However, if you decide to withdraw from the study completely, no more data will be collected about you” (PIL 13)

Other studies lacked clarity confusing the decision to withdraw from the study by asking patients to continue to allow data collection despite the decision not to continue with the study’.

“You and your child can decide not to continue with study at any time but, if you do, we would still like to follow-up your child’s progress” (PIL 41)

### Communicating the importance of retention and follow up

Only eight trials (16%) made any reference to the importance of patient retention or data collection within Patient Information Leaflets. No trials explicitly discussed the problems caused by incomplete data collection. However, eight PIL stated that continuing to provide data was ‘helpful’, ‘valuable’ or ‘important’ when looking at the results and assessing the effect of the treatment.

*“Withdrawing from the study will not affect your child’s medical care*. *We hope, however, that if you do withdraw you would give us a reason and will allow us to continue collecting information from your child’s clinical notes. This will help us when we look at the results of the study” (PIL 6)*

“Even if the original treatment allocation is not possible, we would still ask you to remain in the study as this will give us useful information about the types of patients that may or may not be suited to a particular treatment” (PIL 10)

“It is very important for you to answer all the questions in the questionnaire for us to accurately assess the impact of the treatment upon you” (PIL 13)

“In order to produce reliable results the research centre would like to receive information on you for at least ten years following completion of your treatment” (PIL 16)

### Use of data collected before patient withdrawal

Thirty one of the 50 (62%) trials described options for handling data collected before patient withdrawal in either the PIL or protocol (Table 4). Nineteen of these trials offered patients the option of choosing whether previously collected data was deleted. This was not consistently communicated across the two documents, and was more commonly described within PIL (n = 14, 28%) than protocols (n = 10, 20%). However no Patient Information Leaflets mentioned the impact deleting data might have on the trial and the analysis of results.

### Flexible follow up to increase retention

Only 17 trials (34%) described options for partial data collection to help retain patients (Table 4). Twelve trials (24%) offered the patients the ability to stop all forms of data collection but continue to collect data through routine care appointments, six (12%) offered continued data collection from national records, and six (12%) offered flexibility around trial specific data collection such as questionnaires. However, this information was not communicated consistently across corresponding trial documents.

## Discussion

This study has collated PIL and examined in depth the information provided about retention. It was found that the majority of PIL reiterated the patient’s right to withdraw at any time without penalty and without giving a reason. However, many trials failed to include key information that might reduce unnecessary patient attrition or help assess its impact on the trial. For example, ICH GCP 4.3.4 states that whilst patients are not obliged to give a reason for premature withdrawal, reasonable efforts should be made to ascertain the reason [17] in order to assess potential bias and provide valuable feedback for future trials. 19 (38%) of trials sought to collect withdrawal reasons but only 6 (12%) communicated this to patients demonstrating the need for greater transparency to improve patient awareness and explain why disclosure can be beneficial.

Reasons for attrition vary and may be a result of trial design, patient population or the intervention, but patient withdrawal and loss to follow up are the most common causes [19, 20]. Loss to follow up can be a passive form of withdrawal, where patients fail to complete follow up without formally ending their trial involvement. Trials use a variety of strategies to improve retention such as the use of routine data, reminders and newsletters [15, 19, 21–23]. Many strategies exist to mitigate loss to follow up such as taking multiple contact details, Freephone number for change of address and maintaining contact through newsletters or birthday cards [19]. Yet few strategies address patient withdrawal. Educating participants about the importance of retention could impact both patient withdrawal and loss to follow up rates, and should be implemented alongside other strategies.

Regardless of attrition causes there is an ethical mandate to ensure information provided at consent is consistent and comprehensive. With many participating for altruistic reasons [24–26], a truly informed decision can only be made if communication of the right to withdraw is balanced with information that explains the importance of completing follow up. Yet this information is consistently overlooked by guidance documents rendering patients unaware of its value [27].

The lack of clarity around treatment equipoise and withdrawal terminology should also be a focus for improvement. Only 6% of PIL stated that patients should be willing to have any of the trial treatments and only 30% made a clear distinction between stopping treatment and all study involvement. Few trials explicitly communicated the importance of collecting outcome data if the treatment is not received or stopped, despite half of the cohort wanting to continue to collect data in these circumstances.

Both protocols and PIL were poor at describing follow up options for patients struggling to meet all trial requirements. Whilst we were not able to distinguish between a lack of communication and the constraints of individual outcome measures, data collection through routine appointments and national records poses minimal burden to patients and may be an acceptable alternative to full study withdrawal. Where available, these options along with any flexibility to stop secondary outcome data collection should at least be clarified in trial protocols.

FDA regulated trials are prohibited from making offers to delete patient data collected prior to withdrawal due to concerns about the introduction of bias [28]. Patient rights are fundamental to GCP and should not take precedence over research or the need to develop medical knowledge [29]. However, we recommend the impact of deleting data should be explained but not used to coerce.

The growing list of requirements for PIL content making them longer and less effective [30, 31] may be an important reason why retention content focusses on the basic rights of patients. Recently the HRA issued new guidance on proportionate consent to reduce the information provision for lower risk studies but this does not address the imbalance of retention information observed in our cohort [32]. In the absence of information on the impact of attrition withdrawal can seem inconsequential, increasing the likelihood of patients choosing full study withdrawal before voicing concerns or discussing alternatives with clinical staff. As a minimum we recommend that every PIL includes a statement encouraging patients to talk to clinical staff about ongoing trial participation options if problems emerge. (Table 5)

**Table 5:** Recommendations for retention content within PIL.

Recommendations:
1. Emphasise the need to talk to clinical care team at an early stage if problems emerge. For example, *‘If you develop any concerns over participating in the trial please talk to your doctor or nurse to discuss these and the different options available to you’*.
2. Inform patients that you may ask them why they want to stop taking part and why this information can be helpful.
3. Explain that patients should only take part if they are willing to accept any of the trial treatments.
4. Clearly describe patient withdrawal, making the distinction between premature discontinuation of treatment and stopping all study involvement (study withdrawal).
5. Explain the value of data collection and highlight any options for unobtrusive data collection e.g. through routine records.

Methodological research has assessed the effectiveness of PIL, evaluating the readability of documents [30, 33–36], retention of information by patients [36–38], mode of delivery [36, 39], and presentation format [34, 35, 39] but there remains a paucity of research investigating what information patients receive during the consent process that might influence on retention, adherence and withdrawal. Matsui et al [40] found that presenting patients with more detailed study information reduced recruitment rates but lead to a more certain and sustained participation, reducing the risk of patient attrition. The knowledge of the right to withdraw is frequently recalled [41–43], and has been associated with greater willingness to participate in a trial, helping recruit people who may initially be unsure [44–48].

Explaining the impact of study withdrawal and missing outcome data to patients without being coercive is complex and challenging. Eborall found that patients who stopped their assigned treatment due to an outcome event or side effect expressed disappointment at ending their ‘full participation’ and felt that they were letting the trial down [24]. Many of these patients continued to contribute outcome data to the trial, demonstrating a mismatch between researcher’s and patient’s perceptions of valuable participation and reiterating the need to develop patient education in this area.

### Strengths and weaknesses

Exploring information communicated to patients at consent is a novel approach for addressing retention. This study benefited from dual extraction using a structured approach against UK Health Research Authority and ICH GCP guidelines for PIL content. A comparison of content in both PIL and protocols allowed for more in depth analysis of the communication of trial specific retention approaches to patients. The results are likely to be broadly representative of larger trials undertaken within the UK, although may not be generalizable to early phase trials or public health studies due to the sampling approach.

The research is limited to information documented within PIL and protocols and did not include content from consent discussions. Similarly we did not explore understanding, recall, or relevance of documented information on the decision making process during consent or withdrawal.

### Future research

The impact of retention information within PIL on patient attrition is hypothetical and needs evaluating through SWATs [49], nested Studies Within A Trial. Research should also explore patient perspectives of retention principles and develop acceptable statements to explain how participants can make a valuable contribution, the participation options available and the possible impact of their decision to consent and withdraw from research.

## Conclusion

With many people citing altruistic reasons for participation, improving understanding of treatment equipoise, importance of outcome data collection and continued follow up regardless of treatment adherence, may be an effective strategy to improve retention.

Given the high priority placed on reducing attrition and the lack of balanced information within PIL, further research is warranted to explore the impact of consent information in ongoing trials and develop acceptable, succinct statements to educate patients around retention and withdrawal. Only with this information can patients truly weigh up their own health concerns, the burden of data collection, personal circumstances and any altruistic ambitions in order to make a truly informed decision about initial and ongoing trial participation.

## Abbreviations

CTU: Clinical Trials Unit
HRA: Health Research Authority
HTA: Health Technology Assessment Programme
ICH GCP: International Conference of Harmonisation Good Clinical Practice
PIL: Patient Information Leaflet
RCT: Randomised Control Trial
